# Anti-tumoral effects of a trypsin inhibitor derived from buckwheat *in vitro* and *in vivo*

**DOI:** 10.3892/mmr.2015.3649

**Published:** 2015-04-20

**Authors:** CHONG-ZHI BAI, MA-LI FENG, XU-LIANG HAO, ZHI-JUAN ZHAO, YU-YING LI, ZHUAN-HUA WANG

**Affiliations:** 1 Key Laboratory for Chemical Biology and Molecular Engineering of the Ministry of Education, Institute of Biotechnology, Shanxi University, Taiyuan, Shanxi 030006, P.R. China; 2 Chinese Medicine Hospital of Shanxi Province, Taiyuan, Shanxi 030012, P.R. China

**Keywords:** common buckwheat, trypsin inhibitor, hepatocellular carcinoma, apoptosis, anti-tumor

## Abstract

Native buckwheat, a common component of food products and medicine, has been observed to inhibit cancer cell proliferation *in vitro*. The aim of the present study was to evaluate the *in vitro* and *in vivo* anti-tumoral effects of recombinant buckwheat trypsin inhibitor (rBTI) on hepatic cancer cells and the mechanism of apoptosis involved. Apoptosis in the H22 cell line induced by rBTI was identified using MTT assays, DNA electrophoresis, flow cytometry, morphological observation of the nuclei, measurement of cytochrome C and assessment of caspase activation. It was identified that rBTI decreases cell viability by inducing apoptosis, as evidenced by the formation of apoptotic bodies and DNA fragmentation. rBTI-induced apoptosis occurred in association with mitochondrial dysfunction, leading to the release of cytochrome C from the mitochondria to the cytosol, as well as the activation of caspase-3, -8 and -9. In conclusion, the results of the present study suggested that rBTI specifically inhibited the growth of the H22 hepatic carcinoma cell line *in vitro* and *in vivo* in a concentration-dependent and time-dependent manner, while there were minimal effects on the 7702 normal liver cell line. In addition, rBTI-induced apoptosis in H22 cells was, at least in part, mediated by a mitochondrial pathway via caspase-9.

## Introduction

Buckwheat, an important functional food planted at high latitudes and in colder climates, is a common dietary component in East Asian countries (1). It is high in protein, contains a number of amino acids and is regarded as a popular food globally, particularly in Asia. Buckwheat protein has high biological value due to its well-balanced amino acid composition and its high level of lysine (2,3). Protease inhibitors are widely distributed in nature and are found in numerous animals, plants and microorganisms. They are important in maintaining the balance of proteolytic enzymes *in vivo*. In addition, they regulate endogenous proteases during germination and protect plants against insects and microorganisms (4–6). Of note, the inhibitors have been found to exhibit anti-carcinogenic activities and act as cancer-preventive and anti-inflammatory agents (7–9). Protease inhibitors are capable of inducing the apoptosis of cancer cells *in vitro* and they have drawn attention as potential anti-cancer agents (10). The Bowman-Birk inhibitor family of proteins attained from soybeans are associated with anti-inflammatory and anti-carcinogenic activities (11), and are potentially relevant anti-tumor agents, particularly with regard to colon cancer (12).

Numerous lines of evidence have suggested that protease inhibitors may induce apoptosis in various tumor cell lines; however, the underlying mechanisms of their anti-tumor activity remain to be elucidated. Induction of tumor cell apoptosis is a common mechanism of action of cancer therapeutics (13,14). Caspase-3 is one of the key initiators of apoptosis via the mitochondrial pathway and an essential factor for the activation of the caspase cascade (15–17). Recent studies have revealed that the activation of caspase-9 also induced the activation of the caspase cascade, triggering apoptotic events and inducing cell apoptosis (15,18,19). In addition, another pathway associated with apoptosis is the extrinsic pathway, which is associated with death receptors, including Fas. Adaptor molecules are recruited to the receptors following Fas ligand binding to the Fas death receptor, initiating the program of apoptosis (16,20,21).

Previous studies by our group revealed that a trypsin inhibitor from buckwheat was able to markedly inhibit the proliferation of the IM-9 and K562 cell lines *in vitro* (22,23). In order to elucidate whether the recombinant buckwheat trypsin inhibitor (rBTI) has the same effect *in vivo* and which apoptotic pathway is activated following rBTI treatment, the effect of rBTI treatment on the proliferation of H22 hepatic carcinoma cells was investigated *in vitro* and *in vivo*.

## Materials and methods

### Materials

The H22 hepatic carcinoma cell line and the 7702 normal liver cell line were obtained from the Third Hospital of Shanxi Medical University (Taiyuan, China) and China Radiation Defense and Preservation Institute (Taiyuan, China). For all experiments, 4–6 week-old female BALB/c mice (50 mice; 18–22 g) purchased from the Institute of Laboratory Animal Science, Chinese Academy of Medical Sciences (Beijing, China) were used. All mice were group-housed in plastic cages with stainless-steel grid tops in a room under a 12-h light/dark cycle and fed with distilled water and food. The general health status of the animals was monitored daily. An abdominal injection of the H22 cells (0.2 ml 10^6^ cells/ml with 0.1 ml normal saline) was administered to the BALB/c mice. The cell culture medium was composed of RPMI-1640 (Gibco-BRL, Invitrogen Life Technologies, Carlsbad, CA, USA) supplemented with 10% fetal calf serum (Institute of Hematology, Hangzhou, China), 100 U/ml streptomycin and penicillin solution (100 U/ml) purchased from the Cell Culture Center of the Institute of Basic Medical Sciences, Chinese Academy of Medical Sciences. The cells were incubated at 37°C under humidified conditions with 5% CO_2_. MTT was purchased from Sigma-Aldrich (St. Louis, MO, USA). An Annexin V-fluorescein isothiocyanate Apoptosis Detection kit was obtained from BD Pharmingen (San Diego, CA, USA). An apoptosis DNA Ladder Detection kit was purchased from Nanjing KeyGen Biotech Co., Ltd. (Nanjing, China). A Cytochrome C Detection kit and Caspase-3, -8 and-9 Colorimetric Assay kits were obtained from BioVision (Mountain View, CA, USA). The rBTI was prepared in our laboratory by cloning, expression and one-step affinity purification as described previously (22,23). All other chemicals used were of analytical grade. The current study was approved by the Ethics Committee of Shanxi University (Taiyuan, China).

### Cell viability assay

The cell viability was assessed using an MTT assay. The MTT assay is a colorimetric assay, which measures the percentage of surviving cells. The H22 and 7702 cell lines were separately transferred to quadruplicate wells of 96-well microtiter plates at a density of 5×10^3^ cells/well. The cells were treated with rBTI at concentrations of (6.25, 12.5, 25 and 50 *μ*g/ml for 12, 20 and 24 h, each group investigated in triplicate. A total of 20 *μ*l MTT (5.0 mg/ml) was added and the cells were incubated for 4 h. Following this, 80 *μ*l dimethyl sulfoxide was added. The color intensity was measured using a microtiter plate reader (Model 550; Bio-Rad Laboratories, Inc., Hercules, CA, USA) at 570 nm. The absorbance of the untreated cells was considered to be 100%. The 50% inhibitory concentration was the concentration at which a 50% decrease in the optical density of the drug-treated cells was induced, with respect to untreated cells.

### Morphological observation of nuclei

Following treatment with rBTI (50 *μ*g/ml) for 24 h, the H22 cells were washed with phosphate-buffered saline (PBS; pH 6.7; Sigma-Aldrich) and centrifuged (2,000 ×g), then incubated with 4% paraformaldhyde (Sigma-Aldrich) for 10 min. Subsequently, the cells were collected via centrifugation (2,000 ×g). The cells were stained with 0.1 *μ*g/ml DAPI (Sigma-Aldrich) for 5 min. Fluorescence microscopy was then used to observe the nuclear morphology.

### DNA fragmentation analysis

Following treatment with various concentrations of rBTI (12.5, 25 or 50 *μ*g/ml), a total of 1×10^6^ H22 cells were collected. The DNA of the H22 cells treated with rBTI was extracted according to a procedure described in a previous study by our group (23). The extracted DNA was analyzed on a 1.0% agarose gel (Sigma-Aldrich) with the GeneGenius Bio Imaging system (SynGene, Frederick, MD, USA) and observed under ultraviolet light with an FV1000 fluorescent microscope (Olympus, Tokyo, Japan).

### Flow cytometric analysis of cell apoptosis

Following treatment with rBTI (12.5, 25 or 50 *μ*g/ml) for 24 h, the cells were adjusted to 1×10^6^ in 50 mM PBS at pH 7.6 and centrifuged at 2,000 ×g. A total of 100 *μ*l binding buffer was then added to re-suspend the cells. Annexin V and propidium iodide were added to the cell suspension followed by a 20-min incubation, and the cells were then analyzed using flow cytometry using an FC500 Flow Cytometer (BD Biosciences, Franklin Lakes NJ, USA) (9).

### Measurement of cytochrome C in mitochondria and cytoplasm

Extraction of cytochrome C. Following treatment with rBTI (25 or 50 *μ*g/ml) for 24 h, 1×10^6^ H22 cells were washed with cold PBS. The cells were then centrifuged at 600 ×g for 5 min at 4°C. The supernatant was then removed and the cells were re-suspended in 1.0 ml cytosol extraction buffer. The samples were incubated on ice for 10 min. The homogenate was then transferred to a 1.5-ml microcentrifuge tube and centrifuged at 700 ×g for 10 min at 4°C. The supernatant was then collected into a fresh 1.5-ml tube and centrifuged at 10,000 ×g for 30 min at 4°C. The supernatant was then collected as the cytosolic fraction. The pellet was re-suspended in 0.1 ml mitochondrial extraction buffer mix with protease inhibitors, vortexed for 10 sec and saved as the mitochondrial fraction. After extracting cytochrome C from the mitochondria and cytoplasm, cytochrome C was electro-phoresed using 15% SDS-PAGE.

### Western blot analysis

The protein concentration was determined using a BCA Protein Assay kit (Lanbao Biotechnology, Shanghai, China) according to according to the manufacturer’s protocol. The protein was electrophoresed using 15% SDS-PAGE (as described above) and transferred onto nitrocellulose membranes (Beyotime Institute of Biotechnology, Shanghai, China). Following blocking, the membranes were incubated for 4 h at 37°C with the primary antibody, which was goat anti-mouse monoclonal anti-cytochrome C (1:400; IM 94638; Beyotime Institute of Biotechnology), and the secondary antibody (1:5,000; 572909; Beyotime Institute of Biotechnology), which was goat anti-rat alkaline phosphatase-conjugated immunoglobulin E. The blots were visualized using enhanced chemiluminescence detection reagents (Beyotime Institute of Biotechnology) according to the manufacturer’s instructions. The SynGene GeneTools analysis software-version 3.02.00 (SynGene, Frederick, MD, USA) was used to analyze the images and perform calculations.

### Caspase colorimetric activation assessment

The cells, which were treated with 50 *μ*g/ml rBTI for 24 h, were collected and prepared via incubation with extraction buffer for 30 min and centrifugation at 15,000 ×g for 20 min. Subsequently, the supernatant was collected and the protein concentration was assessed. The protein concentration was determined using a bicinchoninic acid protein assay kit (Beyotime Institute of Biotechnology). The extracts were incubated in a 96-well microtitre plate with three types *p*-nitroanilide (pNA): Asp-Glu-Val-Asp-pNA for caspase-3, Ile-Glu-Thr-Asp-pNA for caspase-8 and Leu-Glu-His-Asp-pNA for caspase-9, respectively, for 2 h at 37°C (BioVision). The absorbance was then measured at 405 nm.

### Animal experiment

The anti-tumoral activity of rBTI against ascites development was evaluated in mice. Following the development of ascites, the peritoneal cavity was washed with 2 ml PBS following treatment with 2.5 mg/100 g pentobarbital (Westang Biotechnology, Shanghai, China). The mice were assigned randomly into five groups: The rBTI treatment groups (four groups) and the control group (n=10 per group). The drug (0.125, 0.25, 0.50 or 1.0 mg/kg; 0.2 ml rBTI) was administered via abdominal injection daily for eight days. The ascites volume was calculated using a burette and the cumulative ascites volume was calculated by adding these values. The total number of viable H22 cells present in the ascites fluid (peritoneal wash) was counted using a hemocytometer.

### Statistical analysis

SPSS 12.0 for Windows (SPSS, Inc., Chicago, IL, USA) was used for statistical analysis. All values are expressed as the mean ± standard error. Comparisons within groups were performed using a one-way analysis of variance and differences between groups were determined using Scheffé’s method. P<0.05 was considered to indicate a statistically significant difference.

## Results

### rBTI inhibits H22 cell proliferation in a dose- and time-dependent manner

**Figure 1:**
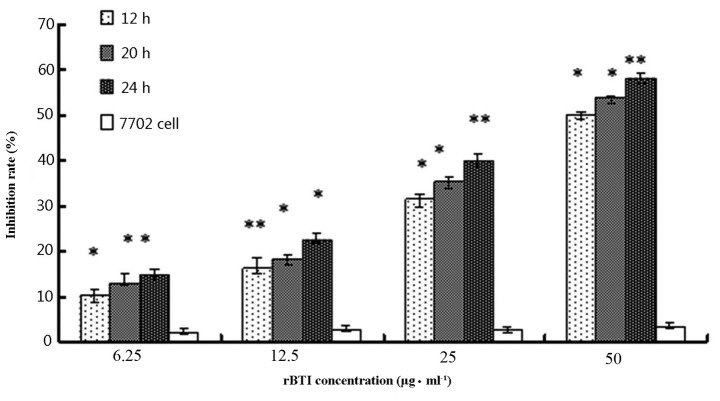
Effects of rBTI treatment on the H22 and 7702 cell lines. Cells were incubated with various concentrations of rBTI. ^*^P<0.05, ^**^P<0.01 compared with 7702 cells. Results are expressed as the mean ± standard error. rBTI, recombinant buckwheat trypsin inhibitor.

The cell growth inhibitory activity of rBTI was assessed using a colorimetric MTT assay, as shown in Fig. 1. The growth inhibition rate of rBTI was 17.8, 27.3, 43.6 and 62.7% at concentrations of 6.25, 12.5, 25 or 50 *μ*g/ml, respectively. The inhibitory effect of rBTI on the proliferation of H22 cells markedly increased as the concentration of rBTI increased. Similarly, the inhibition rate was enhanced with increasing incubation time with the drug. The inhibitory effect of rBTI on the proliferation of H22 cells occurred in a dose-and time-dependent manner; however, there were minimal effects on the 7702 normal liver cell line.

### Nuclear morphological observation

**Figure 2:**
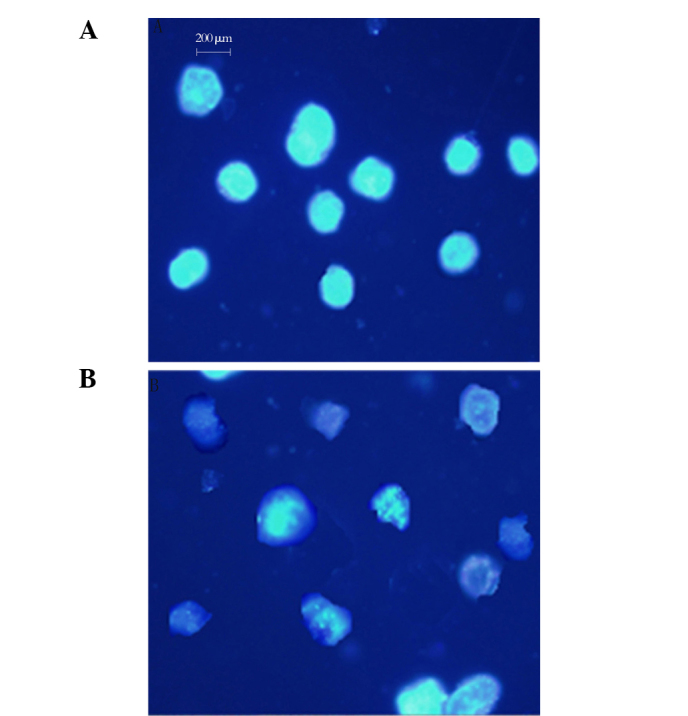
Representative images of normal and apoptotic H22 cells stained with DAPI following incubation without or with 50 *μ*g/ml recombinant buckwheat trypsin inhibitor for 24 h. (A) Control; (B) treated group.

The apoptotic induction effect of rBTI on H22 cells was also investigated. As shown in Fig. 2, following 24 h of 50 *μ*g/ml rBTI treatment, there were significant changes to the cell morphology of H22 cells compared with that of the control cells. The typical characteristics of apoptosis, including membrane disintegration and apoptotic body formation, were also observed (Fig. 2B).

### DNA electrophoresis band analysis

**Figure 3:**
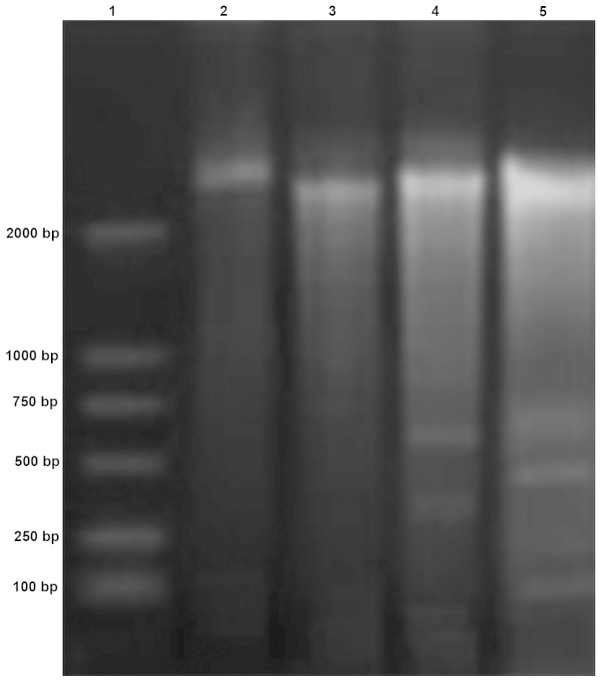
Internucleosomal DNA fragmentation in H22 cells treated with or without rBTI. Lanes 3, 4 and 5: DNA extracts treated with 12.5, 25 and 50 *μ*g/ml rBTI, respectively. Lane 2: DNA extracts without treatment. rBTI, recombinant buckwheat trypsin inhibitor.

The results of the band analysis following DNA electrophoresis revealed that 200-base pair fragments were present following treatment with 50 *μ*g/ml rBTI (Fig. 3). The bands of the DNA electrophoresis presented regularly, similar to a ladder. The band pattern exhibited typical features of apototic cells.

### Flow cytometric analysis

**Figure 4:**
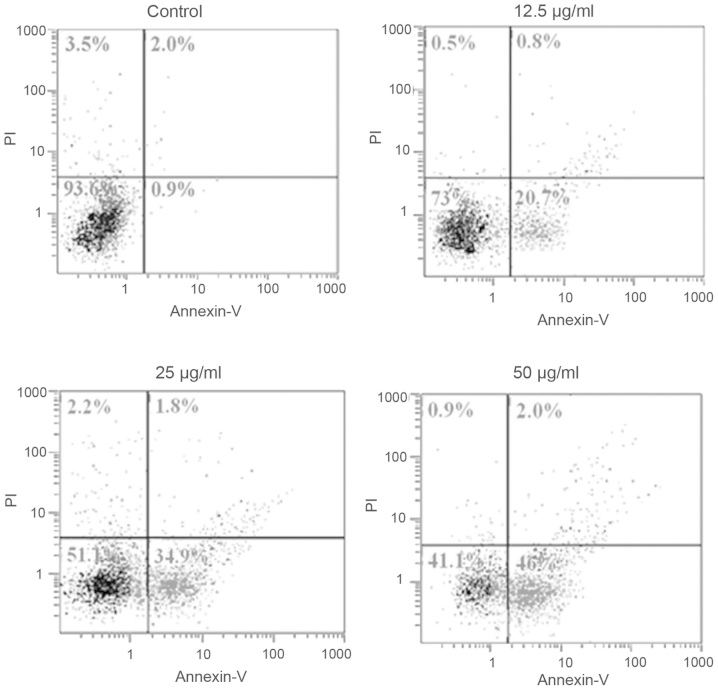
Flow cytometric analysis of apoptotic H22 cells induced by rBTI in a concentration-dependent manner. Cells were incubated with various concentrations of rBTI. rBTI, recombinant buckwheat trypsin inhibitor; PI, propidium iodide.

Cell apoptosis in the H22 cell line was determined by flow cytometry with or without treatment with rBTI at various concentrations (12.5, 25 or 50 *μ*g/ml) for 24 h (Fig. 4). The results revealed that the ratio of apoptotic cells increased with the increase in rBTI concentration. Significant differences were observed among each treatment group and the control.

### Western blot analysis

**Figure 5:**
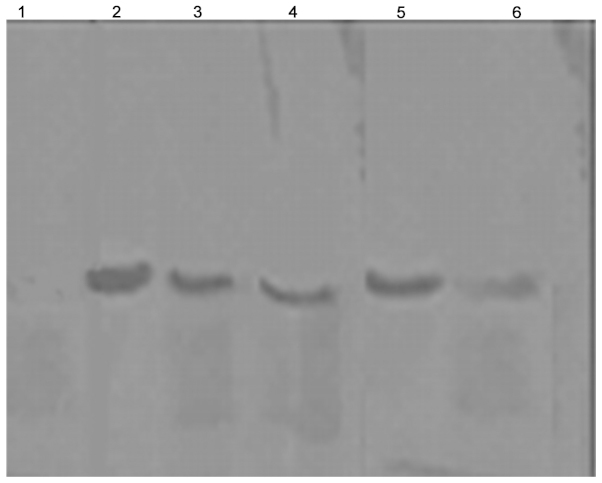
Western blot analysis of cytochrome C. Lanes 1, 3 and 5: Cytochrome C in the cytosol following 0, 25 and 50 *μ*g/ml recombinant buckwheat trypsin inhibitor treatment; lanes 2, 4 and 6: Cytochrome C in the mitochondria following 0, 25 and 50 *μ*g/ml treatment.

Cytochrome C is important in cell apoptosis. It is located in the inter-membrane space between the two membranes. Cytochrome C is released from the mitochondria to the cytosol when the cell undergoes apoptosis. Fig. 5 demonstrates that cytochrome C was released from the mitochondria into the cytoplasm following treatment with rBTI for 24 h and following an increase in the concentration of rBTI from 25 to 50 *μ*g/ml. The quantities of cytochrome C in the cytosol were 54.1 and 74.6% of total cytochrome C, respectively.

### Caspase colorimetric activation assessment

**Figure 6:**
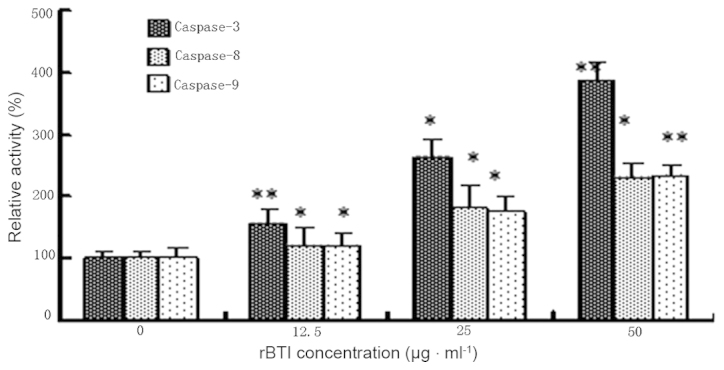
Relative activity of caspases by rBTI in H22 cells. Cell lysates from cells treated with rBTI for 24 h were assayed for *in vitro* caspase-3, -8 and -9 activity using Asp-Glu-Val-Asp-pNA, Ile-Glu-Thr-Asp-pNA and Leu-Glu-His-Asp-pNA, respectively, as substrates. Values are expressed as the mean ± standard error of three independent experiments. ^*^P<0.05, ^**^P<0.01, compared with untreated cells. rBTI, recombinant buckwheat trypsin inhibitor; PNA, *p*-nitroanilide.

As shown in Fig. 6, rBTI-induced apoptosis of H22 cells was predominantly associated with the activities of caspase-3 in a concentration-dependent manner. In addition, caspase-8 and caspase-9 were slightly activated by rBTI. The results supported the hypothesis that rBTI predominantly induced apoptosis in the H22 cell line via the mitochondrial-mediated intrinsic apoptotic pathway involving caspase-3 and caspase-9.

### Inhibition of ascites production in mice following treatment with rBTI

**Figure 7:**
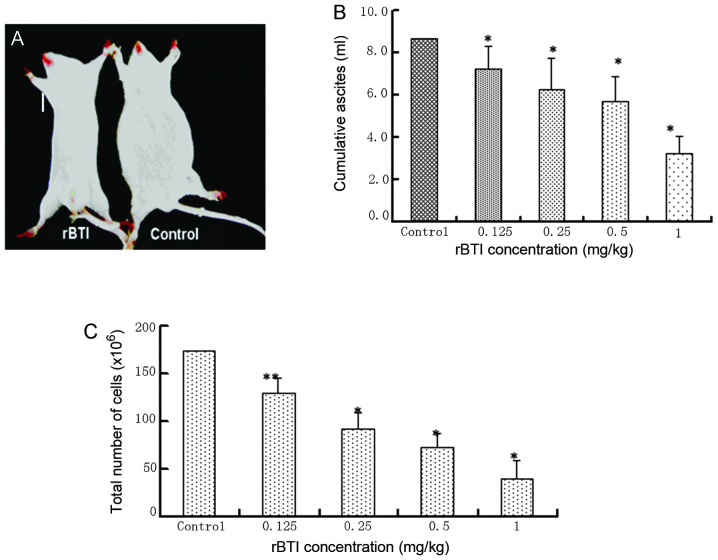
Effect of rBTI on ascites and cancer cell development. Mice inoculated with H22 cells developed ascites and were then randomly assigned to either the control or rBTI treatment group (n=10 per group). (A) All mice in the control group developed overt ascites; however, no macroscopic signs of ascites formation was observed in the 1 mg/kg rBTI-treated mice. (B) The volume of ascites present was recorded. The volume of ascites collected in each animal from the initiation of the treatment was tallied up to provide the cumulative ascites volume. (C) Total number of viable H22 cells present in the ascites fluid (peritoneal wash) was counted using a hemocytometer. ^*^P<0.05, ^**^P<0.01 compared with untreated cells. Results are expressed as the mean ± standard error of ten experiments. rBTI, recombinant buckwheat trypsin inhibitor.

With the development of ascites generation, the abdominal content increased and mice (7 mice with no treatment, 4 mice treated with 0.125 mg/kg, 4 mice treated with 0.25 mg/kg, 3 mice treated with 0.5 mg/kg and 1 mouse treated with 1 mg/kg) died throughout the course of the treatment (8 days). By contrast, mice treated with rBTI at various doses (0.125, 0.25, 0.5 and 1 mg/kg) exhibited a reduction in visible ascites during the entire treatment period of 8 days, particularly those treated with rBTI at the highest concentration of 1 mg/kg (Fig. 7A). The cumulative volume of ascites was evaluated (Fig. 7B). The results revealed that ascites production was significantly suppressed following treatment with rBTI. Similarly, rBTI treatment led to a significant reduction in the quantity of intraperitoneal tumor cells (Fig. 7C).

## Discussion

A preliminary study by our group revealed the efficient *in vitro* anti-tumoral effects of rBTI in K562 cells (23). In the present study, the potent anti-tumoral activity of rBTI was demonstrated *in vitro* and *in vivo*. The cell proliferation inhibition rates of rBTI were 17.8, 27.3, 43.6 and 62.7% at concentrations of 6.25, 12.5, 25 or 50 *μ*g/ml, respectively. The inhibitory effect of rBTI on the proliferation of H22 cells occurred in a dose-dependent and time-dependent manner. rBTI treatment induced caspase activation and also resulted in the translocation of cytochrome C from the mitochondria to the cytosol, as well as the cleavage of DNA, but no effect on the proliferation of normal liver cells was observed. This anti-tumoral activity was partly consistent with the reported biological effects of other inhibitors extracted from buckwheat seeds, including BWI-1 and BWI-2a, which were able to inhibit T-acute lymphoblastic leukemia cell growth *in vitro* (24). The caspase family, which is comprised of aspartate-specific cysteine proteases, is critical in the regulation of apoptosis. The key biochemical pathways of caspase activation are well known (25). Caspase signaling is initiated and propagated by proteolytic autocatalysis and the cleavage of downstream caspases and substrates, including poly adenosine diphosphate ribose polymerase and phospholipase C-γ1 (26). In particular, caspase-3 is one of the key executioners of apoptosis, as it is either partially or completely responsible for the proteolytic cleavage of a number of key proteins (27). The vast majority of cell death signals engage the mitochondrial pathway, where the cysteine protease, caspase-9, is recruited and activated (28). Activation of caspase-9 is mediated by the formation of a macromolecular complex, termed the apoptosome, with the release of cytochrome C from mitochondria (29). In the present study, it was first demonstrated that rBTI increases the release of cytochrome C from the mitochondria. The release of cytochrome C suggested that rBTI induced apoptosis through a mitochondrial pathway (Fig. 5), which is consistent with previous studies (18). In addition, caspase-3, -8 and -9 were activated, which are associated with the mitochondrial intrinsic apoptotic pathway. However, the underlying mechanisms of the induction of mitochondrial dysfunction following treatment with rBTI remain to be elucidated. rBTI may inhibit the synthesis of proteins, which maintain the mitochondrial membrane permeability as a protease inhibitor (30). In addition, it was identified that trypsin certain types of transmembrane protein have a high homology at the DNA level; therefore, rBTI possibly combines with the transmembrane protein and enters the cell, where it induces mitochondrial dysfunction.

The results of the present study also confirmed that rBTI is able to significantly suppress the ascites production in mice. The accumulation of malignant ascites is an important cause of cancer-associated morbidity and mortality in patients with peritoneal metastases (31). In the present study, it was revealed that rBTI was able to significantly suppress ascites formation in H22 tumor-bearing mice. The results clearly demonstrated an effect on the inhibition of proliferation *in vitro* and *in vivo* of rBTI. However, the mechanisms associated with tumor growth inhibition require further investigation.

In conclusion, rBTI, a novel trypsin inhibitor, was shown to exhibit anti-tumoral activity *in vitro* and *in vivo*. rBTI exerted its effects in a dose- and time-dependent manner, while it only had minimal effects on the normal liver cell line 7702. rBTI induced apoptosis by promoting mitochondrial dysfunction, thereby leading to caspase activation. The present study revealed a novel function of rBTI and supported its potential application in treating malignant ascites. The development of a trypsin inhibitor as an anti-tumoral agent requires further investigation.
